# Comparison of Plasma Neurosteroid and Prolactin Levels in Patients with Schizophrenia and Healthy Individuals

**DOI:** 10.1155/2016/3108689

**Published:** 2016-05-10

**Authors:** Forough Riahi, Maryam Izadi-mazidi, Ali Ghaffari, Elham Yousefi, Shahram Khademvatan

**Affiliations:** ^1^Cellular and Molecular Research Center, Ahvaz Jundishapur University of Medical Sciences, Ahvaz, Iran; ^2^Department of Psychiatry, Golestan Educational Hospital, Faculty of Medicine, Ahvaz Jundishapur University of Medical Sciences, Ahvaz, Iran; ^3^Department of Clinical Psychology, Faculty of Humanities, Shahed University, Tehran, Iran; ^4^Department of Medical Biochemistry, Faculty of Medicine, Ahvaz Jundishapur University of Medical Sciences, Ahvaz, Iran; ^5^Department of Parasitology and Mycology, School of Public Health, Tehran University of Medical Sciences, Tehran, Iran; ^6^Cellular and Molecular Research Center and Department of Medical Parasitology and Mycology, Urmia University of Medical Sciences, Urmia 57147 83734, Iran

## Abstract

*Background.* The present study aimed to compare plasma levels of cortisol, testosterone, dehydroepiandrosterone (DHEA), and prolactin in patients with schizophrenia and healthy individuals.* Method.* A total of 100 patients with schizophrenia disorder (69 men and 31 women) and 190 healthy individuals (94 men and 96 women) participated in this cross-sectional study. They were tested for hormone levels and completed demographic questionnaires. Data were analyzed using multivariate analysis of variance (MANOVA) and one-way analysis of variance.* Results.* Serum testosterone level was significantly higher in men with schizophrenia than in healthy men. Women with schizophrenia had a significantly higher level of testosterone and lower level of prolactin compared to healthy women. There were no significant differences in hormone levels across various subtypes of schizophrenia. No significant differences also were observed in hormones levels in patients with first-episode schizophrenia disorder compared to those in patients with recurrent episodes.* Conclusion.* This study indicated that abnormal testosterone and prolactin levels might be associated with pathophysiology of schizophrenia disorder.

## 1. Introduction

Schizophrenia is a major, disabling psychiatric disorder with a devastating impact on patients, their family, and society [1]. This heterogeneous disorder involves a range of cognitive, behavioral, and emotional dysfunctions [2]. The symptoms of schizophrenia fall into three categories: negative symptoms, cognitive dysfunction, and positive symptoms [2]. Negative symptoms of schizophrenia include diminished emotional expression, avolition, alogia, anhedonia, and asociality [2]. The positive symptoms of schizophrenia are hallucinations, delusions, and disorganized speech. Finally, cognitive impairments include disorganized thinking and impaired executive function, working memory, and attention [3].

Its pathophysiology and etiology are complicated and unclear, and the ambiguities in pathogenesis of the disorder underlie our inability to use prevention strategies or effective treatments [4, 5].

Typical onset of schizophrenia is during late adolescence or early adulthood, and there are gender differences in clinical expression of the disorder [2]. According to the fifth edition of the Diagnostic and Statistical Manual of Mental Disorders (2013), general incidence of schizophrenia in females tends to be somewhat lower; the age at onset is later, symptoms tend to be more affect-laden, there are more psychotic symptoms, and psychotic symptoms tend to worsen in later life [2]. Negative symptoms and disorganization are less frequent in females and social functioning remains better preserved [3]. Gender differences in various dimensions of this disorder and the association between onset of schizophrenia and reproductive age led some to suggest involvement of hormonal factors in pathophysiology of schizophrenia [6].

Neuroendocrinological studies have suggested that dysfunction of the hypothalamic-pituitary-adrenal and/or hypothalamic-pituitary-gonadotropin axis may contribute to the pathophysiology of schizophrenia [7–9].

The relationship of the serum levels of testosterone [7–13], prolactin [7, 8], dehydroepiandrosterone (DHEA) and/or its sulfate conjugate (DHEA-S) [8, 11–13], ACTH [9], cortisol [9, 12, 13], progesterone [7, 9], gonadotropins [7], and estradiol [8, 13] with schizophrenia have been evaluated by some studies and diverse findings have been obtained. Since more knowledge about the pathogenesis of the disorder would result in more effective prevention and treatment strategies and investigation of correlation between schizophrenia and plasma hormones levels has received little attention in Iran, the present study aimed to compare plasma levels of cortisol, testosterone, dehydroepiandrosterone (DHEA), and prolactin in patients with schizophrenia and healthy individuals in Ahvaz, Southwest Iran.

## 2. Materials and Methods

### 2.1. Subjects

This cross-sectional study was conducted over a period of 12 months from 2014 to 2015. One hundred patients diagnosed with schizophrenia (69 men and 31 women) and 190 healthy individuals (94 men and 96 women) participated in the study.

The patients were selected from the psychiatric yard of Golestan Educational Hospital affiliated to Jundishapur University of Medical Sciences in Ahvaz, Iran. The diagnosis of schizophrenia disorder was performed by two psychiatrists following the DSM-IV-TR criteria.

The control group consisted of blood donors who were tested at the laboratory in Jundishapur University of Medical Sciences. They were assessed by both a clinical psychologist and a psychiatrist and had no history of schizophrenia disorder.

None of the two groups had any other major psychiatric disorders or neurological diseases. Patients were not under treatment (ECT or taking psychiatric drugs) during the last 6 months.

To participate, subjects had to agree to comply with the requirements of study, and after describing the procedures and purposes of the study, written informed consents were obtained. The study was approved by the ethical committee of the university (number ajums.REC.1392.350).

A 5 mL blood sample was taken from each subject for hormone assays. Each subject was also asked to complete a questionnaire to obtain demographic data about ethnicity, gender, age, level of education, marital status, and employment.

ELISA method was performed for determination of testosterone, dehydroepiandrosterone (DHEA), cortisol, and prolactin (Monobind, USA) concentration. Final results were recorded by ELISA reader and in the form of optic absorbance (OD = 450). Quantitative examination of the samples was done by drawing standard curve and through optic absorbance of positive and negative controls and determined consistencies.

### 2.2. Statistical Analysis

Data were analyzed using multivariate analysis of variance (MANOVA) and one-way analysis of variance. The probability level of 0.05 was accepted as statistically significant. Statistical analyses were carried out using SPSS version 16.

## 3. Results

The mean age was 37.67 (±11.37) in women with schizophrenia disorder and 24.53 (±4.27) in healthy women. Mean ages of schizophrenic men and healthy men were 35.8 (±10) and 25.5 (±4.9), respectively. Frequencies of the participants' demographic features are listed in Table 1.

Plasma levels of testosterone, DHEA, cortisol, and prolactin hormones in normal controls and patients with schizophrenia were compared using multivariate analysis of variance (MANOVA). There were significant differences between patient and healthy women in testosterone (*F* = 7.9, *p* < 0.001) and prolactin (*F* = 231.5, *p* = 0.005). A comparison of all four hormones is shown in Table 2.

There was a significant difference between patient and healthy men in testosterone (*F* = 141.67, *p* < 0.001). A comparison of all four hormones is shown in Table 3.

Plasma levels of hormones in patients with different subtypes of schizophrenia disorder were compared using one-way analysis of variance.

The difference in serum levels of testosterone, DHEA, cortisol, and prolactin hormones among women with different subtypes was not statistically significant (all *p* > 0.05, Table 4).

The mean values for all four hormones were not found to differ markedly among men with different subtypes of schizophrenia disorder (all *p* > 0.05, Table 5).

There were no significant differences in hormones levels in patients with first-episode schizophrenia disorder compared to those in patients with recurrent episodes (*p* > 0.05, Tables 6 and 7).

## 4. Discussion

The purpose of the present study was to compare plasma neurosteroid (cortisol, testosterone, dehydroepiandrosterone (DHEA)), and prolactin levels in patients with schizophrenia and healthy individuals.

Our findings showed that plasma levels of testosterone, in male and female patients with schizophrenia, were significantly higher than those in the healthy controls. This is in line with some previous studies, even though the studies are somewhat different. Oades and Schepker [12] found that female patients with schizophrenia had higher testosterone level than matched healthy controls. In the studies conducted by Mason et al. [11, 14], male inpatients with schizophrenia had higher serum testosterone level when compared to patients with mood disorders.

Testosterone regulates the actions of a wide range of neurotransmitters. Investigations support the association between increased level of testosterone and aggression. In addition, free testosterone in the CSF has been found to associate with aggression, suspiciousness, sensation seeking and monotony avoidance, and reduced socialization [1]. Moreover, it is reported that anabolic steroids can cause a variety of adverse psychiatric effects, including aggressive behavior, mood and psychotic disturbances, and psychological dependence, particularly in higher doses [1]. Testosterone exposure may explain the earlier age of onset and greater incidence of schizophrenia disorder among men [15].

However, some evidence does not support a role for elevated testosterone in schizophrenia disorder. For example, Brown et al. [10] report no difference between patients with schizophrenia and healthy individuals with respect to testosterone secretion. And Akhondzadeh et al. [7] indicated that male patients with predominant and nonpredominant negative symptoms had lower plasma levels of testosterone and free testosterone when compared with healthy controls. Other studies [8, 9, 13] reported a significant negative correlation between serum testosterone level and negative symptoms in the male inpatients with predominant negative symptoms of schizophrenia.

We also found that female (but not male) patients with schizophrenia disorder had significantly lower plasma level of prolactin compared to healthy women. This is consistent with the finding that the secretion of prolactin is inhibited by dopamine neurons located in the tuberoinfundibular section of the hypothalamus [1]. Therefore, it can be decreased by increased release of dopamine in schizophrenia disorder [16].

Our finding could also be explained based on the hypothesis of hypoestrogenism. According to this hypothesis, estrogen deficiency could be involved in pathogenesis of schizophrenia in women [6]; on the other hand, estrogen may increase the serotonin-stimulated release of prolactin [1]. Therefore, decrement in serum prolactin may be associated with low estrogen level in female patients. We did not measure estrogen level and thus we did not identify its association with prolactin in our patient women.

In the study by Luchins et al. [17], no relationship was found between prolactin level and psychopathology. However, Akhondzadeh et al. [7] indicated that plasma level of prolactin in male patients with the predominant negative symptoms of schizophrenia was significantly higher than that in the aged matched normal men. And Ko et al. [8] did not find any significant correlation between the serum prolactin level and the severity of negative symptoms in male patients with schizophrenia.

With respect to the serum DHEA level, we could not find any difference between two groups. Previous studies showed somewhat contradictory results, that is, equivalent levels [18–20] or lower level [21] in patients with schizophrenia compared to healthy controls. Other studies showed higher dehydroepiandrosterone sulfate (DHEA-S) level in young male patients with schizophrenia [12] and in first-episode schizophrenia subjects [22]. Shirayama et al. [9] and Ko et al. [8] did not find correlation between DHEA(S) and the severity of negative symptoms in male patients with schizophrenia.

In the current study, we did not find significant difference between patients and healthy individuals in plasma level of cortisol. Some previous studies [23, 24] reported high levels of serum cortisol in patients with schizophrenia. Shirayama et al. [9] showed positive correlation between plasma level of cortisol and the severity of negative symptoms of schizophrenia. And Meltzer et al. [25] found that baseline plasma cortisol levels were significantly higher in schizophrenic patients compared to healthy controls.

We propose some reasons for the differences between the results of the present and previous investigations. A number of prior studies [7–9, 13] included patients who were taking antipsychotic and it has been reported that chronic treatment with antipsychotic drugs decreases plasma level of testosterone [26–28] and increases prolactin plasma level [29]. Moreover, differences between the results of our study and those of prior studies may also be explained by differences in illness characteristics between patient groups. In the studies by Akhondzadeh et al., Ko et al., Shirayama et al., and Goyal et al., patients with predominantly negative symptoms of schizophrenia were included [7–9, 13].

In conclusion, the current study suggests that reproductive hormones and function of hypothalamic-pituitary-gonadotropin axis could be considered as a biological marker in pathophysiology of schizophrenia disorder.

The main strength of the present study is that patients were not taking antipsychotic, because neuroleptic agents may influence pituitary-gonadal hormones.

Several limitations should, however, be taken into account. It has been reported that stress, diet, exercise, and sexual activity affect serum testosterone levels [8]. It was possible that these factors are involved in determination of the serum testosterone levels in each patient in this study. We did not measure other related hormone levels, including estrogen, gonadotropin-releasing hormone, luteinizing hormone, and adrenocorticotropic hormone, and therefore we did not know their associations with symptoms of schizophrenia. We also did not compare patients with predominant negative symptoms and patients with nonpredominant negative symptoms in plasma neurosteroid and prolactin levels. Thus, these are suggested in future studies.

## Figures and Tables

**Table 1:** Frequencies of the participants' demographic features.

Demographic variable	Frequency *N* (%)	
	Patients group	Healthy individuals
Gender		
Male	69 (69%)	94 (49.5%)
Female	31 (31%)	96 (50.5%)
Marital status		
Single	64 (64%)	137 (72%)
Married	27 (27%)	53 (28%)
Divorced/widowed	9 (9%)	0 (0%)
Education		
Grade school	70 (70%)	22 (11.5%)
12 years/high school	19 (19%)	19 (10%)
University degree	11 (11%)	149 (78.5%)
Ethnicity		
Fars	39 (39%)	72 (37.89%)
Arab	24 (24%)	57 (30%)
Lor	31 (40%)	46 (24.21%)
Other	6 (6%)	15 (7.89%)

**Table 2:** Comparison using multivariate analysis of variance (MANOVA) of serum hormones levels in women with schizophrenia disorder and healthy women.

Mean (±standard deviation)				*F*_1,95_	*p* value
		Patients with schizophrenia	Healthy individuals		
Female	Testosterone	2.55 (±1.54)	0.9 (±0.84)	7.9	<0.001
	DHEA	(1.6 ± 1.7)	1.46 (±1.35)	2.4	0.12
	Prolactin	4 (±3.5)	6.39 (±4.23)	231.5	0.005
	Cortisol	13.6 (±6.03)	13.9 (±6.1)	0.04	0.8

**Table 3:** Comparison using multivariate analysis of variance (MANOVA) of serum hormones levels in men with schizophrenia disorder and healthy men.

Mean (±standard deviation)				*F*_1,171_	*p* value
		Patients with schizophrenia	Healthy individuals		
Male	Testosterone	8.11 (±4.32)	4.28 (±0.93)	141.67	<0.001
	DHEA	1.11 (±1.46)	1.69 (±1.58)	0.15	0.69
	Prolactin	4.88 (±3.72)	4.44 (±3.51)	2.38	0.12
	Cortisol	13.6 (±6.7)	13.55 (±6.35)	0.12	0.72

**Table 4:** Comparison using one-way analysis of variance of serum hormones levels in women with different subtypes of schizophrenia disorder.

Mean (±standard deviation)						*F*_4,25_	*p* value
	Women with paranoid subtype (*n* = 18)	Women with catatonic subtype (*n* = 2)	Women with residual subtype (*n* = 1)	Women with undifferentiated subtype (*n* = 8)	Women with disorganized subtype (*n* = 1)		
Testosterone	2.63 (±0.47)	2.65 (±0.63)	2.48 (±0)	2.58 (±1.9)	1 (±0)	0.33	0.8
DHEA	1.73 (±1.92)	0.64 (±0.78)	0.9 (±0)	1.39 (±1.34)	1.06 (±0)	0.25	0.9
Prolactin	4.17 (±3.8)	6.45 (±6.4)	3 (±0)	3.42 (±2.69)	2 (±0)	0.35	0.8
Cortisol	13.11 (±6.33)	15 (±5.65)	22 (±0)	13.12 (±6.1)	11 (±0)	0.55	0.6

**Table 5:** Comparison using one-way analysis of variance of serum hormones levels in men with different subtypes of schizophrenia disorder.

Mean (±standard deviation)						*F*_4,25_	*p* value
	Men with paranoid subtype (*n* = 46)	Men with catatonic subtype (*n* = 5)	Men with residual subtype (*n* = 2)	Men with undifferentiated subtype (*n* = 10)	Men with disorganized subtype (*n* = 4)		
Testosterone	8.14 (±4.6)	7.42 (±5.5)	6.95 (±0.07)	7.66 (±1.75)	7.4 (±3.58)	0.09	0.9
DHEA	1.19 (±1.47)	0.35 (±0.64)	3.1 (±4.23)	0.92 (±1.22)	0.41 (±0.53)	1.56	0.19
Prolactin	4.86 (±3.8)	3.4 (±1.5)	5.5 (±4.94)	6.3 (±4.4)	5 (±2.58)	0.54	0.7
Cortisol	13.3 (±6.7)	14.2 (±9)	12.5 (±6.36)	13.1 (±6.26)	15.75 (±7.5)	0.14	0.9

**Table 6:** Comparison using multivariate analysis of variance (MANOVA) of serum hormones levels in women with first-episode schizophrenia disorder and recurrent episodes.

	Women with first-episode schizophrenia (*n* = 11)	Women with recurrent episodes (*n* = 23)	*F*_1,27_	*p* value
Testosterone	2.45 (±1.53)	2.62 (±1.23)	2.37	0.13
DHEA	2.07 (±2.52)	1.44 (±1.31)	0.913	0.3
Prolactin	4.02 (±4.23)	4.1 (±3.3)	0.003	0.9
Cortisol	10.55 (±6.71)	14.71 (±5.44)	3.88	0.059

**Table 7:** Comparison using multivariate analysis of variance (MANOVA) of serum hormones levels in men with first-episode schizophrenia disorder and recurrent episodes.

	Men with first-episode schizophrenia (*n* = 20)	Men with recurrent episodes (*n* = 45)	*F*_1,60_	*p* value
Testosterone	8.11 (±3.59)	8.13 (±4.6)	0.04	0.826
DHEA	1.37 (±1.66)	1.02 (±1.4)	0.889	0.350
Prolactin	5.2 (±3.47)	4.86 (±3.89)	0.069	0.794
Cortisol	13.55 (±7.3)	13.61 (±6.55)	0.004	0.952
